# Data demonstrating the influence of the latent storage efficiency on the dynamic thermal characteristics of a PCM layer

**DOI:** 10.1016/j.dib.2017.04.005

**Published:** 2017-04-11

**Authors:** D. Mazzeo, G. Oliveti, N. Arcuri

**Affiliations:** Department of Mechanical, Energy and Management Engineering (DIMEG), University of Calabria, P. Bucci 46/C, 87036 Rende, CS, Italy

## Abstract

Dynamic thermal characteristics, for each month of the year, of PCM layers with different melting temperatures and thermophysical properties, in a steady periodic regime, were determined (Mazzeo et al., 2017 [1]). The layer is subjected to climatic conditions characterizing two locations, one with a continental climate and the second one with a Mediterranean climate. This data article provides detailed numerical data, as a function of the latent storage efficiency, including monthly average daily values: of the latent energy fraction, of the decrement factors of the temperature, of the heat flux and of the energy, and of the time lags of the maximum and minimum peaks of the temperature and of the heat flux.

**Specifications Table**

**Table:** 

Subject area	*Energy Engineering*
More specific subject area	*Building physics, Building envelope materials, Latent thermal storage*
Type of data	*Table (Microsoft Excel file format), Figure (PDF file format)*
How data was acquired	*Finite difference numerical calculation model*
Data format	*Technical data sheet, analysed and processed output data*
Experimental factors	Commercial data sheets have provided the PCM thermophysical properties
	The climatic data were generated with the TRNSYS software for the localities of Turin and Cosenza
Experimental features	*The dynamic thermal parameters in the presence of one or more bi-interfaces in the different months of the winter, intermediate and summer air-conditioning seasons were calculated*
Data source location	*Cosenza, Italy*
Data accessibility	*Data is within this article.*

**Value of the data**

• Data show the correlation between the dynamic thermal parameters and the latent storage efficiency, namely the fraction of the PCM thickness subject to phase change.
• Data regard various commercial PCM layers with different melting temperatures, two localities and the winter, intermediate and summer seasons.
• Data are useful to choose which type of PCM is suitable in building external wall application.

## 1 Data

“Supplementary Table 1” presents a database of a set of dynamic thermal parameters of different types of PCMs. The boundary conditions of the PCM layer are those that characterize the external walls of air-conditioned buildings.

Each material has different melting temperature and thermophysical properties.

The data are related to the different months of the year for the two locations of Turin and Cosenza.

## 2 Experimental design, materials and methods

The calculation procedure, based on an explicit finite difference numerical model which resolves the equation of conduction in solid phase and liquid phase and the equation of thermal balance at the bi-phase interfaces at the melting temperature, has been performed by the followed steps:

1) Creation of the mathematical model and calculation algorithm.
2) Definition of a new set of dynamic parameters.
3) PCM thermophysical properties retrieval from commercial data sheets [2], [3], [4], [5], [6].
4) Generation of the monthly average daily values of the climatic data of the two localities with the TRNSYS software [7].
5) Calculation of the dynamic parameters for five PCMs, two localities and all the months of the year.
6) Construction of the dynamic parameters trends as a function of the latent storage efficiency.

The details of the methodology are presented in [1].

“Supplementary Fig. 1.pdf” shows, ​for the different PCMs, the monthly average daily values of the latent energy fraction Λ_L_, and of the decrement factors of temperature f_T_, of heat flux f_Φ_ and of energy f_E_ depending on the latent storage efficiency ε_L_. In “Supplementary Fig. 2.pdf”, for each of the different PCMs, the values of the time lag of maximum peak and of the minimum peak of the temperature ${\Delta t}_{T^{\max}}$ and ${\Delta t}_{T^{\min}}$ and of the heat flux $\Delta t_{\Phi^{\max}}$ and $\Delta t_{\Phi^{\min}}$ as a function of the latent storage efficiency ε_L_ are reported. In each image of Figs. 1 and 2, the obtained values of a dynamic parameter, in the two locations and in different months of the year, upon variation of the latent storage efficiency are presented for a given PCM. For such representations, we used more pointers: in all the columns of Fig. 1 and in the first three columns of Fig. 2, the triangle and circle symbols identify the dynamic parameter values relative, respectively, to Turin and to Cosenza; in the fourth column of Fig. 2, the pointers were differentiated based upon the air conditioning season, as the functional dependence of Δt_Φ^max^_ on the efficiency of latent storage is different in the two air conditioning seasons.

Concerning the dynamic parameters in Fig. 1, all the trends can be represented by second grade polynomial functions, while regarding the dynamic parameters in Fig. 2 all the trends can be represented by a first grade polynomial function.

Concerning the decrement factor of the temperature f_T_ trend, it is necessary to specify that, during the months when the constant peak time fraction is different to zero, it is subject to a change of concavity with a reduction of the values. This behaviour concerns only PCMs S15 and LATEST20T. ​Transparency document.Supplementary material Transparency data associated with this article can be found in the online version at http://dx.doi.org/10.1016/j.dib.2017.04.005.
