# Supplementary material for: Data demonstrating the influence of the latent storage efficiency on the dynamic thermal characteristics of a PCM layer
Source: Data Brief. 2017 Apr 11;12:274–6. doi: 10.1016/j.dib.2017.04.005 (PMC5403787; doi:10.1016/j.dib.2017.04.005)
Supplement: Supplementary file 1 — Supplementary material [file mmc1.docx]

The authors whose names are listed immediately below certify that they have NO affiliations with or involvement in any organization or entity with any financial interest (such as honoraria; educational grants; participation in speakers’ bureaus; membership, employment, consultancies, stock ownership, or other equity interest; and expert testimony or patent-licensing arrangements), or non-financial interest (such as personal or professional relationships, affiliations, knowledge or beliefs) in the subject matter or materials discussed in this manuscript. We have no conflicts of interest to disclose. We confirm that neither the manuscript nor any parts of its content are currently under consideration or published in another journal. We confirm that the manuscript has been read and approved by all named authors and that there are no other persons who satisfied the criteria for authorship but are not listed. We further confirm that the order of authors listed in the manuscript has been approved by all of us. We confirm that we have given due consideration to the protection of intellectual property associated with this work and that there are no impediments to publication, including the timing of publication, with respect to intellectual property. In so doing we confirm that we have followed the regulations of our institutions concerning intellectual property. We understand that the Corresponding Author is the sole contact for the Editorial process (including Editorial Manager and direct communications with the office). He is responsible for communicating with the other authors about progress, submissions of revisions and final approval of proofs. We confirm that we have provided a current, correct email address which is accessible by the Corresponding Author and which has been configured to accept email from domenico.mazzeo@unical.it. Author names: Mazzeo Domenico Oliveti GiuseppeArcuri Natale
