# Supplementary material for: Data demonstrating the influence of the latent storage efficiency on the dynamic thermal characteristics of a PCM layer
Source: Data Brief. 2017 Apr 11;12:274–6. doi: 10.1016/j.dib.2017.04.005 (PMC5403787; doi:10.1016/j.dib.2017.04.005)
Supplement: Supplementary file 2 — Supplementary material [file mmc2.pdf]

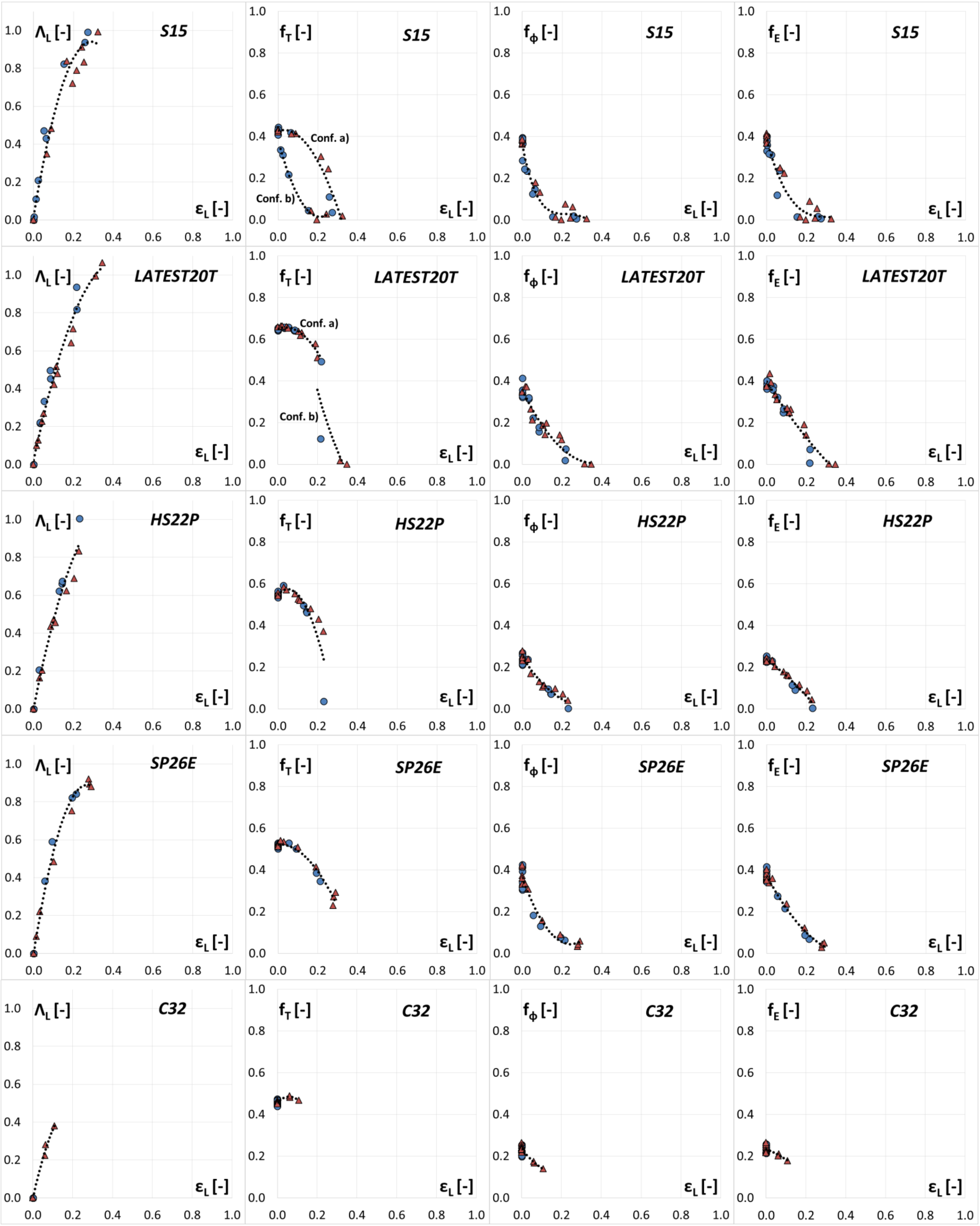

Figure 1 – Latent energy fraction Λ_L and decrement factors of temperature f_T, heat flux f_Φ and energy f_E as a function of the latent storage efficiency ε_L for different PCMs

Turin   Cosenza
