# Supplementary material for: Data demonstrating the influence of the latent storage efficiency on the dynamic thermal characteristics of a PCM layer
Source: Data Brief. 2017 Apr 11;12:274–6. doi: 10.1016/j.dib.2017.04.005 (PMC5403787; doi:10.1016/j.dib.2017.04.005)
Supplement: Supplementary file 3 — Supplementary material [file mmc3.pdf]

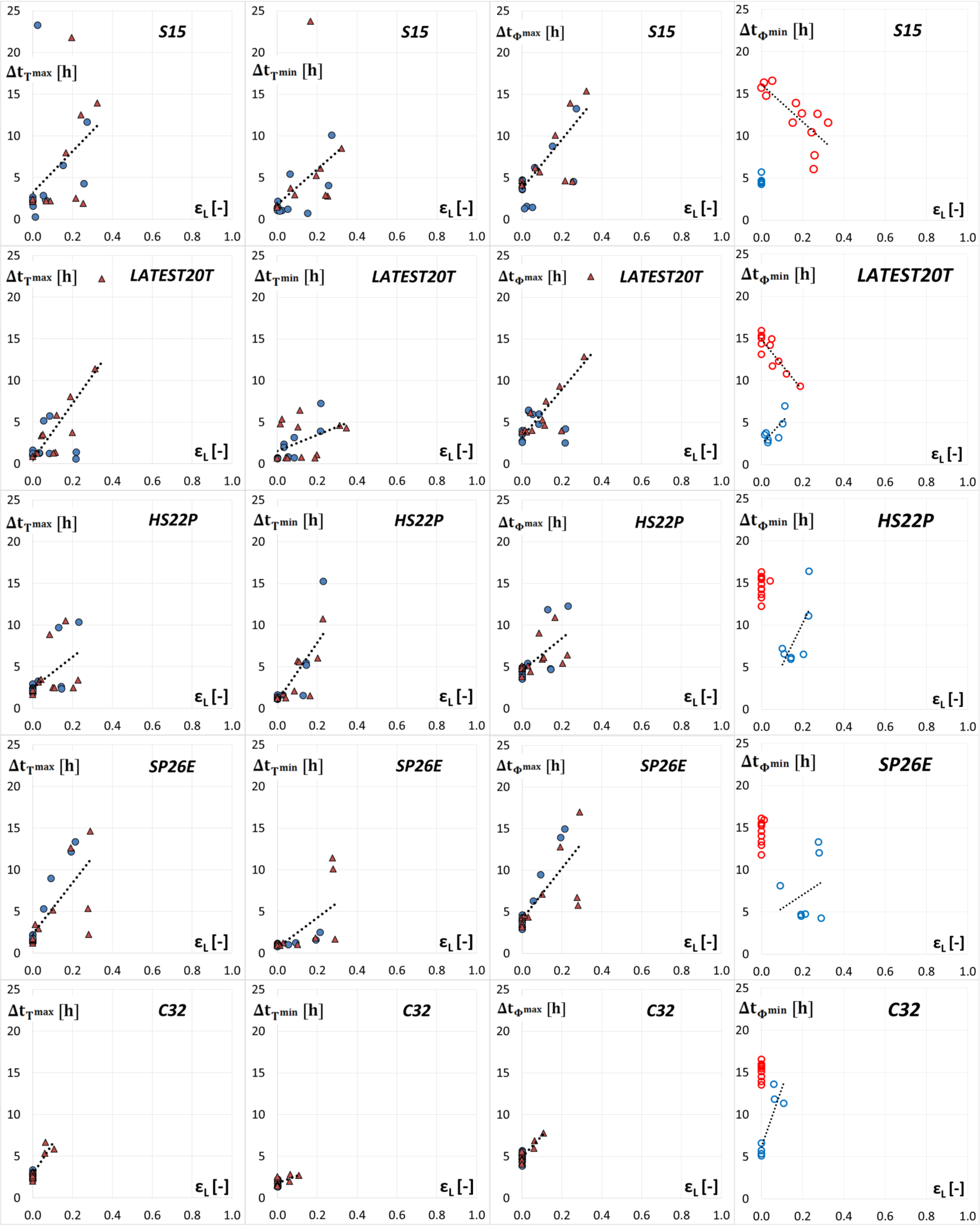

Figure 2 - Time lag of the maximum and mimum peak of the temperature $\Delta t_{T^{max}}$ and $\Delta t_{T^{min}}$ and of the heat flux $\Delta t_{\phi^{max}}$ and $\Delta t_{\phi^{min}}$ as a function of latent storage efficiency ε_L for the different PCMs.

▲Turin ●Cosenza ○Winter period ○Summer period
